# Bictegravir/emtricitabine/tenofovir alafenamide (B/F/TAF) in treatment-naïve and treatment-experienced people with HIV: 12-month virologic effectiveness and safety outcomes in the BICSTaR Japan cohort

**DOI:** 10.1371/journal.pone.0313338

**Published:** 2025-01-08

**Authors:** Yoshiyuki Yokomaku, Katsuji Teruya, Dai Watanabe, Tomoyuki Endo, Rumi Minami, Nao Taguchi, Tali Cassidy, Andrea Marongiu, David Thorpe, Takuma Shirasaka, Shinichi Oka

**Affiliations:** 1 Clinical Research Center, NHO Nagoya Medical Center, Nagoya, Japan; 2 AIDS Clinical Center, National Center for Global Health and Medicine, Tokyo, Japan; 3 AIDS Medical Center, NHO Osaka National Hospital, Osaka, Japan; 4 Department of Hematology, Hokkaido University Hospital, Sapporo, Japan; 5 Internal Medicine, Clinical Research Institute, NHO Kyushu Medical Center, Fukuoka, Japan; 6 Medical Affairs-HIV, Gilead Sciences K.K., Tokyo, Japan; 7 Real-World Evidence, Gilead Sciences Europe Ltd, Uxbridge, United Kingdom; 8 Global Medical Affairs, Gilead Sciences Europe Ltd, Uxbridge, United Kingdom; NIH: National Institutes of Health, UNITED STATES OF AMERICA

## Abstract

BICSTaR (BICtegravir Single Tablet Regimen) is an ongoing, observational cohort study assessing the virologic effectiveness and safety of bictegravir/emtricitabine/tenofovir alafenamide (B/F/TAF) in treatment-experienced (TE) and treatment-naïve (TN) people with HIV across 14 countries over 24 months. We present 12-month outcomes from participants in the BICSTaR Japan cohort. Retrospective and prospective data were pooled from people with HIV aged ≥20 years receiving B/F/TAF within routine clinical care in Japan. Outcomes included virologic effectiveness (primary endpoint; HIV-1 RNA <50 copies/mL), CD4 count, CD4/CD8 ratio, drug-related adverse events (DRAEs), persistence, and patient-reported outcomes (prospective TN cohort only). Overall, 200 participants were enrolled and included in the 12-month analysis population (150 retrospective, 50 prospective; 116 TN and 84 TE). Most participants were male at birth (99%); median age was 34 years in TN and 45 years in TE participants. At 12 months, virologic effectiveness was high: 92% (90/98) of TN and 95% (72/76) of TE participants had HIV-1 RNA <50 copies/mL (missing = excluded analysis). Median (quartile [Q]1, Q3) CD4 cell count increased by +202.0 (126.0, 311.0) cells/μL in TN (p<0.001) and +11.0 (−60.0, 87.0) cells/μL in TE (p = 0.380) participants. Through 12 months, DRAEs were reported by 13% (25/200) of all participants (16% [18/116] TN, 8% [7/84] TE); diarrhea, weight gain, and headache were the most common. Most DRAEs were mild in severity and no severe DRAEs were reported. One TN participant (<1%; 1/116) and two TE participants (2%; 2/84) discontinued B/F/TAF due to DRAEs (macrocytic anemia, vertigo, diarrhea, and headache). Treatment persistence at 12 months exceeded 98% in both TN and TE participants. In prospective TN participants, improvements in bothersome symptom count and quality-of-life measures were observed. B/F/TAF demonstrated high levels of virologic effectiveness and tolerability in people with HIV treated as part of routine clinical care in Japan.

## Introduction

In Japan, the incidence of HIV is relatively low, with 34,421 people estimated to be living with HIV in 2022 [1]. The number of newly diagnosed cases has decreased over recent years to 884 cases in 2022 [1]. Furthermore, the introduction of combination antiretroviral treatment (ART) has seen the mortality rate of people with HIV decrease throughout Asia more widely [2].

There have been rapid advances in HIV treatment in Japan over the past four decades; however, with these improvements, new challenges are emerging [3]. The population of people with HIV in Asia as a whole is aging [4], with most people with HIV in Japan now benefiting from long-term treatment [5]. Optimal ART must maintain long-term virologic suppression, have a good safety profile, and possess a high barrier to resistance [6–8]. Comorbidities and concomitant medication use are high in people with HIV in Japan and will increase further with advancing age [3, 9, 10]; therefore, factors such as pill burden and drug–drug interactions are important considerations in this aging population when choosing ART regimens [6–8, 11]. Single-tablet, fixed-dose combinations of ART taken once daily have simplified the management of HIV, and have been associated with improved adherence compared with multi-tablet regimens [12, 13].

Two- and three-drug ART regimens are recommended for the initial treatment of HIV by the current international and local Japanese HIV guidelines [7, 8, 14]. Bictegravir, emtricitabine, and tenofovir alafenamide (B/F/TAF) is a three-drug regimen co-formulated as a single tablet. It has demonstrated safety and efficacy in numerous studies in both treatment-naïve (TN) and treatment-experienced (TE) people with HIV [15–22]. B/F/TAF is approved in multiple countries, including Japan, where it is recommended as a first-line treatment for HIV [7, 8, 14, 23]. Although several studies have confirmed the real-world safety and effectiveness of B/F/TAF in routine clinical practice [24–32], evidence specific to the Japanese HIV population is very limited.

BICtegravir Single Tablet Regimen (BICSTaR) is an ongoing, multinational, prospective and retrospective, observational study with a planned follow-up of 24 months, designed to assess the real-world virologic effectiveness and safety of B/F/TAF in TN and TE people with HIV. The study has enrolled 2379 TN and TE people with HIV across 14 countries in 5 observational cohorts (Europe, Canada, Israel, Asia [Taiwan, South Korea, Singapore], and Japan). Pooled 12-month data from 12 countries [33] and data from the BICSTaR Asia cohort (Singapore, the Republic of Korea, and Taiwan) [34] have been reported previously.

Here, we report data on the virologic effectiveness and safety of B/F/TAF through 12 months in 200 people with HIV in the BICSTaR Japan cohort.

## Methods

### Study design

In-depth methodology for the BICSTaR program has been published previously [33]. For this 12-month analysis, participants were enrolled between 1 June 2020 and 23 April 2021 and data were collected between 1 June 2020 and 8 March 2023 in people with HIV receiving B/F/TAF as part of routine clinical care at five participating sites in Japan: NHO Osaka National Hospital, NHO Nagoya Medical Center, National Center for Global Health and Medicine, NHO Kyushu Medical Center, and Hokkaido University Hospital. The BICSTaR Japan cohort will be followed for 24 months in the main BICSTaR study.

Data were collected prospectively in participants who initiated or switched to B/F/TAF at study entry (prospective cohort) and both retrospectively and prospectively in participants who were already taking B/F/TAF prior to study entry (retrospective cohort). Data were acquired from clinical records, hospital files, clinic visits, electronic medical records, and validated patient-reported outcome (PRO) questionnaires (prospective cohort only) and entered into electronic case report forms. The authors did not have access to information that could identify individual participants during or after data collection.

The BICSTaR protocol was approved by an independent ethics committee at each study center. The study was conducted in accordance with Ethical Guidelines for Medical and Health Research Involving Human Subjects. Follow-up visits complied with the standard practice of each site, based on the decision of the treating physician. No additional diagnostic or laboratory monitoring procedures were required.

### Participants

Eligible participants were people with HIV aged ≥20 years either initiating (prospective cohort) or currently receiving (retrospective cohort) B/F/TAF as part of routine clinical care in accordance with the Japan Package Insert [35]. Individuals who participated in any interventional clinical trial without prior consent from the Study Sponsor, or in any HIV-related drug joint studies, were excluded. Written informed consent was received from all participants. Participant selection was at the discretion of the investigator, based on the Japan Package Insert and inclusion/exclusion criteria; where possible, enrollment was done in a consecutive manner.

### Treatment

Eligible participants received B/F/TAF (50 mg/200 mg/25 mg) in accordance with the Japan Package Insert and local treatment guidelines in Japan.

### Study endpoints and assessments

The primary endpoint was evaluation of the virologic effectiveness of B/F/TAF, measured by the proportion of participants with virologic suppression (HIV-1 RNA <50 copies/mL) at month 12. Secondary endpoints included the proportion of participants with HIV-1 RNA <50 copies/mL at months 3 and 6; change from baseline to month 12 in CD4 count and CD4/CD8 ratio, and the cumulative incidence of adverse events (AEs), serious AEs (SAEs), and drug-related AEs (DRAEs) at month 12. AEs were coded using the Medical Dictionary for Regulatory Activities (MedDRA V25.1).

Exploratory endpoints included reasons for initiating ART in TN participants, reasons for switching to B/F/TAF in TE participants, persistence, treatment discontinuations, reasons for discontinuation and change from baseline to month 12 in weight, body mass index (BMI), lipid profiles (total cholesterol, high-density lipoprotein, low-density lipoprotein, triglycerides) and renal function (estimated glomerular filtrate rate [eGFR]).

PRO measures were collected from prospective participants only. Data are presented for the prospective TN cohort only due to the small sample size of the prospective TE cohort (n = 8). Treatment satisfaction was measured using the HIV Treatment Satisfaction status (HIVTSQs) questionnaire [36]. HIVTSQs scores range from 0 to 60, with higher scores indicating higher treatment satisfaction. Overall bothersome symptom count was measured using the HIV-Symptom Index (HIV-SI) questionnaire [37]; mental and physical health-related quality of life were measured using the 36-item Short-Form Health Survey (SF-36v2) Mental Component Summary (MCS) and Physical Component Summary (PCS) scores [38].

### Statistical analysis

For the primary endpoint analysis, a sample size of 200 was deemed appropriate to detect a proportion of participants with virologic suppression (HIV-1 RNA <50 copies/mL) of ≥90% with a power of 80% for one-sample proportion (Score z test: target proportion = 90%, smallest detectable proportion = 95.4%).

An in-depth explanation of statistical analyses across the BICSTaR program has been described previously [33]. Descriptive statistics were employed for demographics and outcomes measures in the TN and TE cohorts.

The primary endpoint analysis at 12 months was a missing-equals-excluded (M = E) analysis in participants with ≥1 HIV-1 RNA value within the 12-month time window (≥275 to ≤548 days). Participants who discontinued the study and/or B/F/TAF before the 12-month window, or who had missing data, were excluded (no imputation). M = E analyses were also completed at 3 and 6 months.

A treatment discontinuation-equals-failure (D = F) sensitivity analysis was also performed to evaluate virologic effectiveness at 3, 6, and 12 months. At month 12, this analysis included participants with ≥1 HIV-1 RNA value within the 12-month window and participants who discontinued B/F/TAF before the 12-month window (imputed as having HIV-1 RNA ≥50 copies/mL).

Median changes from baseline to 12 months in CD4 count, CD4/CD8 ratio, weight, BMI, lipids, eGFR (calculated using the Cockcroft–Gault formula [39]), and PROs were assessed using 95% two-sided p-values and/or confidence intervals and Sign test, signed rank test, Wilcoxon test, or Student t-test. For these analyses, *p* < 0.05 indicates statistical significance. For sample sizes <20 participants, statistical tests were not conducted.

Persistence with B/F/TAF at month 12 was defined as still being in the study and having received B/F/TAF for ≥275 days (i.e. the lower bound of the 12-month visit window) and was calculated as the number of participants remaining on B/F/TAF within the 12-month visit window divided by the total number of participants in the study (excluding participants who discontinued the study before the 12-month time window with no evidence of B/F/TAF discontinuation).

Baseline was defined as the point at which participants initiated or switched to B/F/TAF; for retrospective participants, baseline was prior to study entry. Among retrospective participants, the median duration of B/F/TAF use prior to the start of the study was 6.2 and 6.9 months in the TN and TE cohorts, respectively. There was potential for overestimation of virologic effectiveness and persistence and underestimation of AE incidence in the retrospective cohort (i.e., immortal time bias) as individuals who discontinued due to treatment failure prior to study initiation were excluded. To evaluate the presence of immortal time bias, virologic suppression rates were numerically compared between the retrospective and prospective cohorts.

SAS software, version 9.4 (SAS institute Inc., Cary, NC) was used to perform statistical analyses.

## Results

### Baseline demographic and disease characteristics

**Fig 1:**
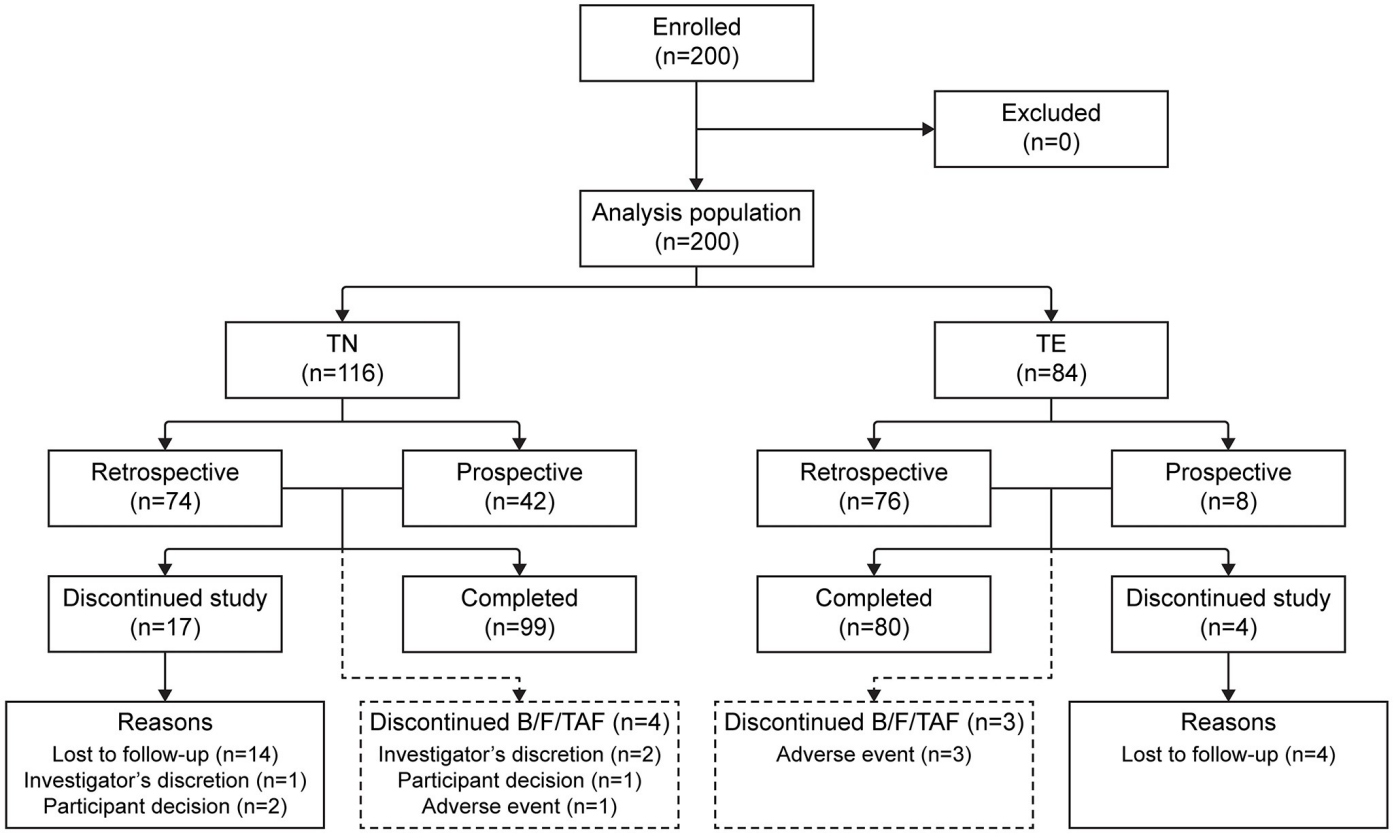
Participant flow diagram. B/F/TAF, bictegravir/emtricitabine/tenofovir alafenamide; TE, treatment-experienced; TN, treatment-naïve.

Overall, 200 people with HIV were enrolled in the BICSTaR Japan cohort and included in the analysis population (150 retrospective, 50 prospective; 116 TN and 84 TE) as of the data cut-off (04 May 2023; Fig 1). For this analysis, median follow-up time was 18.0 months in TN and TE participants. Overall, median B/F/TAF treatment duration was 23.5 and 23.9 months in TN and TE participants, respectively, at the end of the study. However, for the purposes of this 12-month analysis, only laboratory results and AEs reported until the upper bound of the 12-month visit window (≤548 days) were included.

Participant demographics and baseline characteristics are shown in Table 1. Most participants were male at birth (99%) and Asian (99%). On average, TN participants were younger than TE participants (median age 34.0 vs. 45.0, respectively). Most participants had ≥1 comorbidity (80%), the most common of which were neuropsychiatric disorders and hyperlipidemia, and more than one-third were receiving ≥1 concomitant non-ART medication (35%). Baseline characteristics of the retrospective and prospective cohorts are shown in S1 Table.

**Table 1:** Baseline demographics and disease characteristics by ART status.

	TN (n = 116)	TE (n = 84)
Sex,^a^ n (%)		
Male	116 (100)	82 (97.6)
Female	0	2 (2.4)
Age		
Median (Q1, Q3), years	34.0 (28.0, 44.5)	45.0 (39.0, 50.0)
<50 years, n (%)	104 (89.7)	60 (71.4)
≥50 years, n (%)	12 (10.3)	24 (28.6)
Median (Q1, Q3) weight,^b^ kg	63.7 (56.7, 70.0)	69.1 (61.6, 76.0)
Min, Max	41.7, 107.8	48.8, 99.0
Median (Q1, Q3) BMI,^b^ kg/m^2^	22.3 (19.8, 24.0)	23.4 (21.6, 26.3)
Min, Max	15.3, 35.9	17.5, 35.4
Race/ethnicity, n (%)		
White	1 (0.9)	0
Asian	114 (98.3)	84 (100)
Asian and White	1 (0.9)	0
Median (Q1, Q3) eGFR, mL/min/1.73 m^2^	116.0 (99.1, 130.2)	101.2 (85.2, 122.1)
Comorbidities/co-infections, n (%)		
None	32 (27.6)	18 (21.4)
1	33 (28.4)	15 (17.9)
2	22 (19.0)	13 (15.5)
≥3	29 (25.0)	38 (45.2)
Most common		
Hyperlipidemia	4 (3.4)	27 (32.1)
Neuropsychiatric	15 (12.9)	15 (17.9)
Hypertension	7 (6.0)	13 (15.5)
Diabetes mellitus	3 (2.6)	8 (9.5)
Chronic hepatitis B/C	2 (1.7)	8 (9.5)
HIV-1 RNA viral load^c^		
Median (Q1, Q3), log_10_ copies/mL	4.85 (4.46, 5.30)	1.28 (1.28, 1.28)
<50 copies/mL, n (%)	0	66 (88.0)
>100,000 copies/mL, n (%)	43 (38.4)	3 (4.0)
Median (Q1, Q3) CD4 count, cells/μL^d^	297.5 (173.0, 480.0)	572.0 (427.0, 714.0)
Median (Q1, Q3) CD4/CD8 ratio^e^	0.30 (0.20, 0.48)	0.90 (0.60, 1.09)
Late HIV diagnosis, n (%)^f^		
CD4 <350 cells/μL^g^	74 (64.9)	-
CD4 <200 cells/μL^g^	32 (28.1)	-
Concomitant non-ART medications at baseline,^h^ n (%)		
None	54 (61.4)	35 (44.9)
1	13 (14.8)	12 (15.4)
2	9 (10.2)	12 (15.4)
≥3	12 (13.6)	19 (24.4)
Median (Q1, Q3) number of previous ART regimens^i^	-	2.0 (1.0, 3.0)
Prior ART regimens (taken just prior to B/F/TAF),^j^ n (%)		
INSTI	-	49 (58.3)
DTG		20 (23.8)
EVG		18 (21.4)
RAL		11 (13.1)
NNRTI	-	17 (20.2)
PI	-	15 (17.9)
TDF	-	6 (7.1)
TAF	-	59 (70.2)
History of prior virologic failure,^k^ n (%)	-	2 (2.5)
Time from HIV diagnosis to B/F/TAF initiation, median (Q1, Q3) days	57.0 (36.0, 103.0)	-

^a^ Sex was defined by the individual.

^b^ Sample size: TN n = 101, TE n = 66.

^c^ Sample size: TN n = 112, TE n = 75.

^d^ Sample size: TN n = 110, TE n = 75.

^e^ Sample size: TN n = 108, TE n = 74.

^f^ Sample size: n = 114.

^g^ And/or ≥1 AIDS-defining event at baseline.

^h^ Sample size: TN n = 88, TE n = 78.

^i^ Sample size: n = 83.

^j^ Includes combination and single-agent therapy.

^k^Sample size: n = 79.

ART, antiretroviral therapy; B/F/TAF, bictegravir/emtricitabine/tenofovir alafenamide; BMI, body mass index; CD, cluster of differentiation; DTG, dolutegravir; eGFR, estimated glomerular filtration rate; EVG, elvitegravir; INSTI, integrase strand transfer inhibitor; NNRTI, non-nucleoside reverse transcriptase inhibitor; PI, protease inhibitor; Q, quartile; RAL, raltegravir; TAF, tenofovir alafenamide; TDF, tenofovir disoproxil fumarate; TE, treatment-experienced; TN, treatment-naïve.

In the TN cohort, the most common reason for initiating B/F/TAF was related to local treatment guidelines (98%) while in the TE cohort, the most common reason for switching to B/F/TAF from previous regimens was simplification of ART (58%) (S2 Table). At baseline, 3% (5/200) of all participants had evidence of ≥1 primary resistance mutation (nucleoside reverse transcriptase inhibitor [NRTI], 2%; non-NRTI [NNRTI], 1%; protease inhibitor [PI], 1%; integrase strand transfer inhibitor [INSTI], 0%) [S3 Table].

### Virologic effectiveness

**Fig 2:**
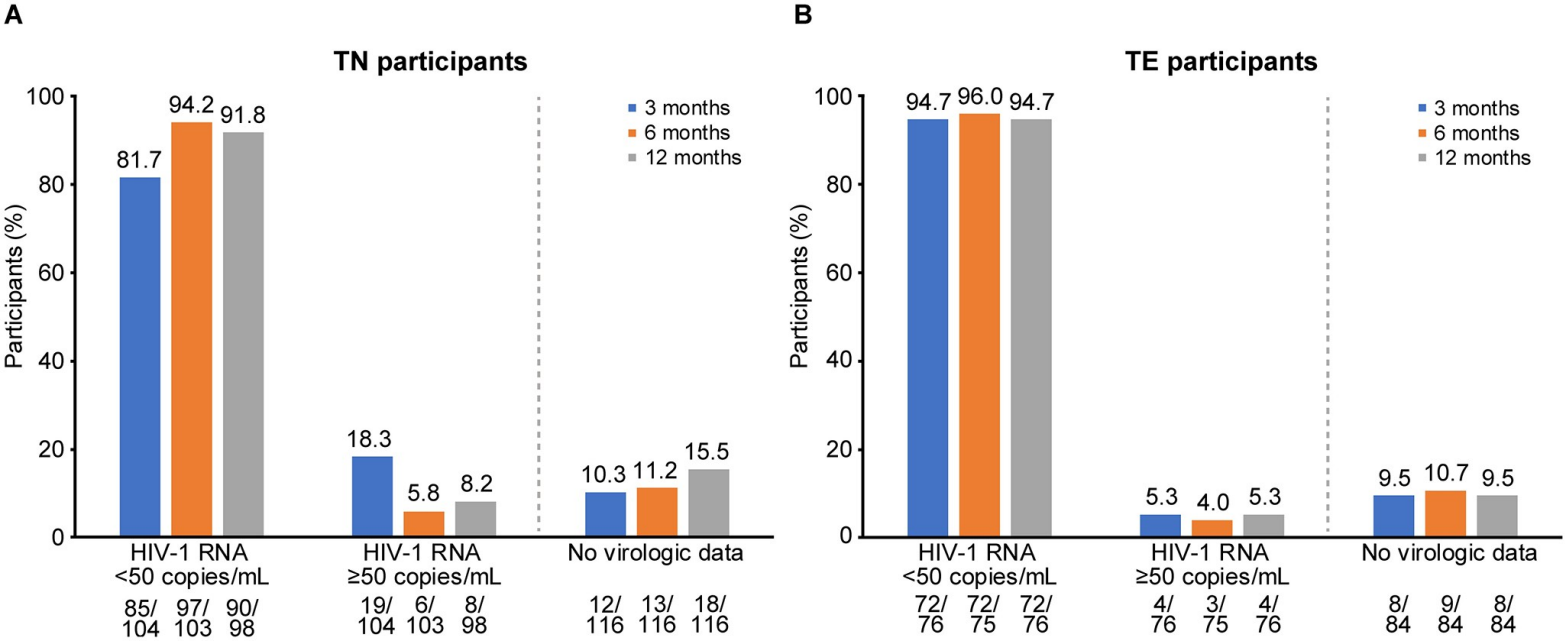
Virologic outcomes at 3, 6, and 12 months (M = E analysis). (A) TN and (B) TE participants. At 12 months, of the 26 participants with no virologic data, 1 TN participant discontinued B/F/TAF before the 12-month time window (but remained in the study), 7 (6 TN and 1 TE) participants withdrew from the study before the 12-month time window (but remained on B/F/TAF), 1 TN participant discontinued B/F/TAF and withdrew from the study before the 12-month time window and 17 (10 TN and 7 TE) remained on B/F/TAF before the 12-month time window and had their last visit ≥275 days from B/F/TAF initiation. M = E, missing-as-excluded; TE, treatment-experienced; TN, treatment-naïve.

Virologic outcomes at 3, 6 and 12 months are shown in Fig 2. At 12 months, high rates of virologic suppression were observed in both TN and TE cohorts. In an M = E analysis, 92% (90/98) of TN and 95% (72/76) of TE participants had HIV-1 RNA <50 copies/mL. Eight TN participants and four TE participants had HIV-1 RNA ≥50 copies/mL (M = E analysis); of these, one TE participant had HIV-1 RNA ≥200 copies/mL. In a D = F analysis, 90% (90/100) of TN and 95% (72/76) of TE participants had HIV-1 RNA <50 copies/mL, respectively (S4 Table). At 12 months, HIV-1 RNA viral load and the proportion of participants with HIV-1 RNA <50 copies/mL was similar in retrospective and prospective cohorts (*p* = 0.7136 for TN participants; S1 Fig).

### Immunological outcomes

**Fig 3:**
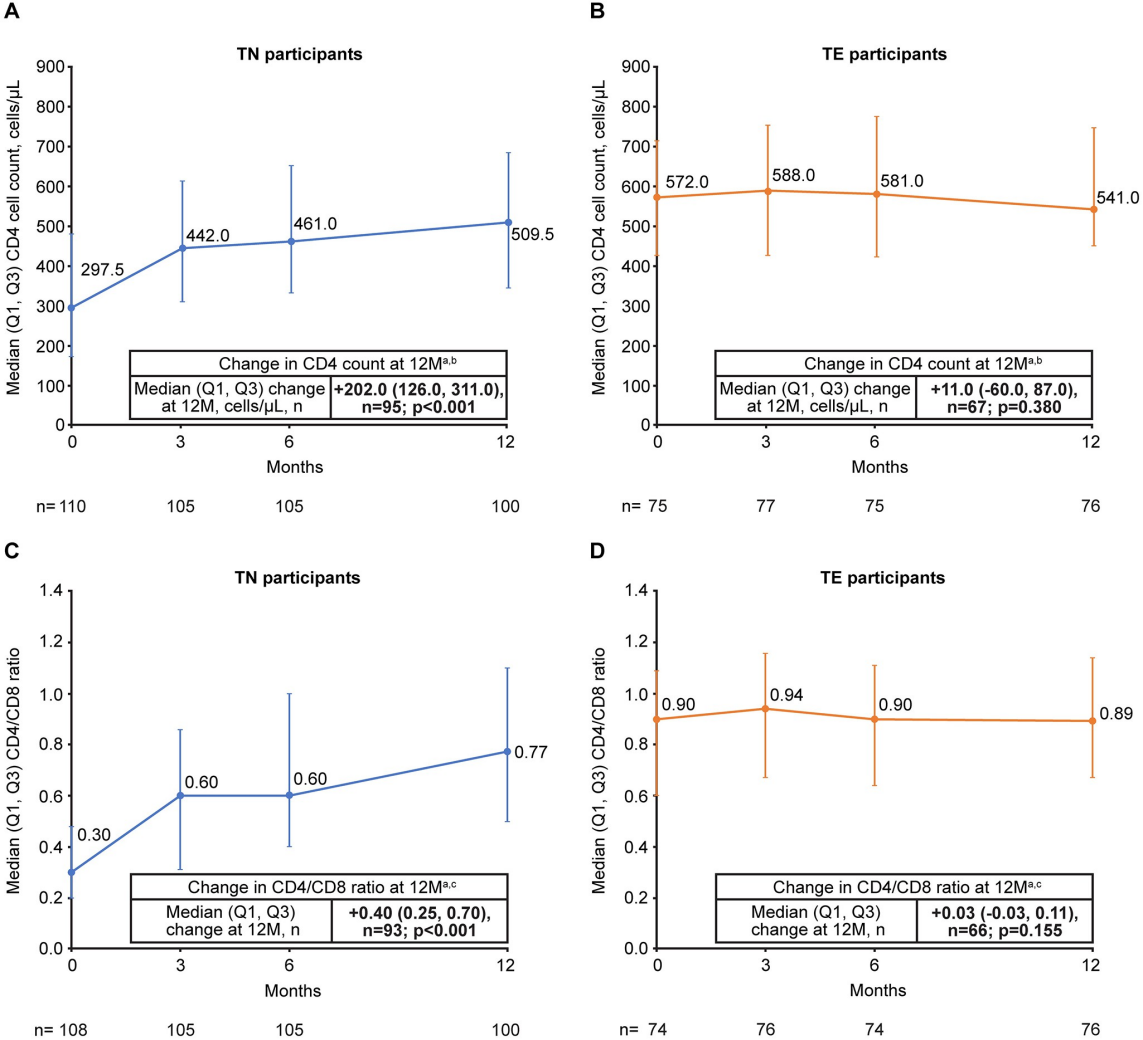
Change in CD4 count in (A) TN and (B) TE participants and change in CD4/CD8 ratio in (C) TN and (D) TE participants from baseline to 12 months. ^a^ In participants with data available at baseline and at 12 months. ^b^ *p*-values calculated using the Signed Rank test. ^c^ *p*-values calculated using the Sign test. CD, cluster of differentiation; M, months; Q, quartile; TE, treatment-experienced; TN, treatment-naïve.

There was a statistically significant increase in median CD4 cell count and CD4/CD8 ratio from baseline to 12 months in TN participants; these measures remained stable in TE participants (Fig 3). Median (quartile [Q] 1, Q3) CD4 count change was +202.0 (126.0, 311.0) cells/μL (*p <* 0.001) in TN participants and +11.0 (−60.0, 87.0) cells/μL (*p* = 0.380) in TE participants. Median (Q1, Q3) CD4/CD8 ratio change was +0.40 (0.25, 0.70) in TN (*p* < 0.001) and +0.03 (−0.03, 0.11) in TE participants (*p* = 0.155). Changes in CD4 count and CD4/CD8 ratio were similar between retrospective and prospective cohorts at 12 months.

### Safety and tolerability

AEs reported through 12 months are summarized in Table 2. Up to 12 months, AEs were reported by 67% (134/200) of all participants; 71% (82/116) of TN participants and 62% (52/84) of TE participants. Incidence of AEs was similar in the retrospective and prospective TN cohorts; however, a lower proportion of retrospective TE participants reported AEs compared with prospective TE participants (61% [46/76] vs 75% [6/8]). The majority of AEs were unrelated to B/F/TAF. DRAEs were reported by 13% (25/200) of all participants; 16% (18/116) of TN participants and 8% (7/84) of TE participants. The most commonly reported DRAEs were diarrhea (n = 5), weight gain (n = 5), and headache (n = 4). Most DRAEs were mild in severity and no severe DRAEs were reported. In participants reporting weight gain as a DRAE who had weight data available at baseline and 12 months (n = 3), the range in baseline weight was 56.0–68.2 kg and weight change from baseline to 12 months was 14.2–24.1 kg. By month 12, three participants discontinued B/F/TAF due to DRAEs (macrocytic anemia, vertigo, diarrhea, and headache). SAEs were reported by 12% (23/200) of all participants; 12% (14/116) of TN participants and 11% (9/84) of TE participants. One TN participant reported a drug-related SAE of immune reconstitution inflammatory syndrome. There were no B/F/TAF discontinuations due to hepatic, renal, bone, or weight gain DRAEs, and no deaths were reported.

**Table 2:** Adverse events reported through 12 months overall and by ART status.

Summary of AEs, n (%)	All (n = 200)	TN (n = 116)	TE (n = 84)
Any AE	134 (67.0)	82 (70.7)	52 (61.9)
Drug-related AEs	25 (12.5)	18 (15.5)	7 (8.3)
Gastrointestinal disorders	6 (3.0)	4 (3.4)	2 (2.4)
Weight gain	5 (2.5)	5 (4.3)	0
Headache	4 (2.0)	3 (2.6)	1 (1.2)
Psychiatric disorders	2 (1.0)	2 (1.7)	0
Urticaria	2 (1.0)	1 (0.9)	1 (1.2)
Reproductive system and breast disorders	2 (1.0)	1 (0.9)	1 (1.2)
Glycosylated hemoglobin increased	1 (0.5)	0	1 (1.2)
Cognitive disorder	1 (0.5)	1 (0.9)	0
Macrocytic anemia	1 (0.5)	0	1 (1.2)
Palpitations	1 (0.5)	1 (0.9)	0
Vertigo	1 (0.5)	1 (0.9)	0
Immune reconstitution inflammatory syndrome	1 (0.5)	1 (0.9)	0
Herpes zoster	1 (0.5)	1 (0.9)	0
Muscular weakness	1 (0.5)	1 (0.9)	0
Any SAE	23 (11.5)	14 (12.1)	9 (10.7)
Discontinued B/F/TAF due to DRAEs	3 (1.5)	1 (0.9)	2 (2.4)
Macrocytic anemia	1 (0.5)	0	1 (1.2)
Vertigo	1 (0.5)	1 (0.9)	0
Diarrhea	1 (0.5)	0	1 (1.2)
Headache	1 (0.5)	1 (0.9)	0
Deaths	0	0	0

AE, adverse event; ART, antiretroviral therapy; B/F/TAF, bictegravir/emtricitabine/tenofovir alafenamide; DRAE, drug-related AE; SAE, serious adverse event; TE, treatment-experienced; TN, treatment-naïve.

**Fig 4:**
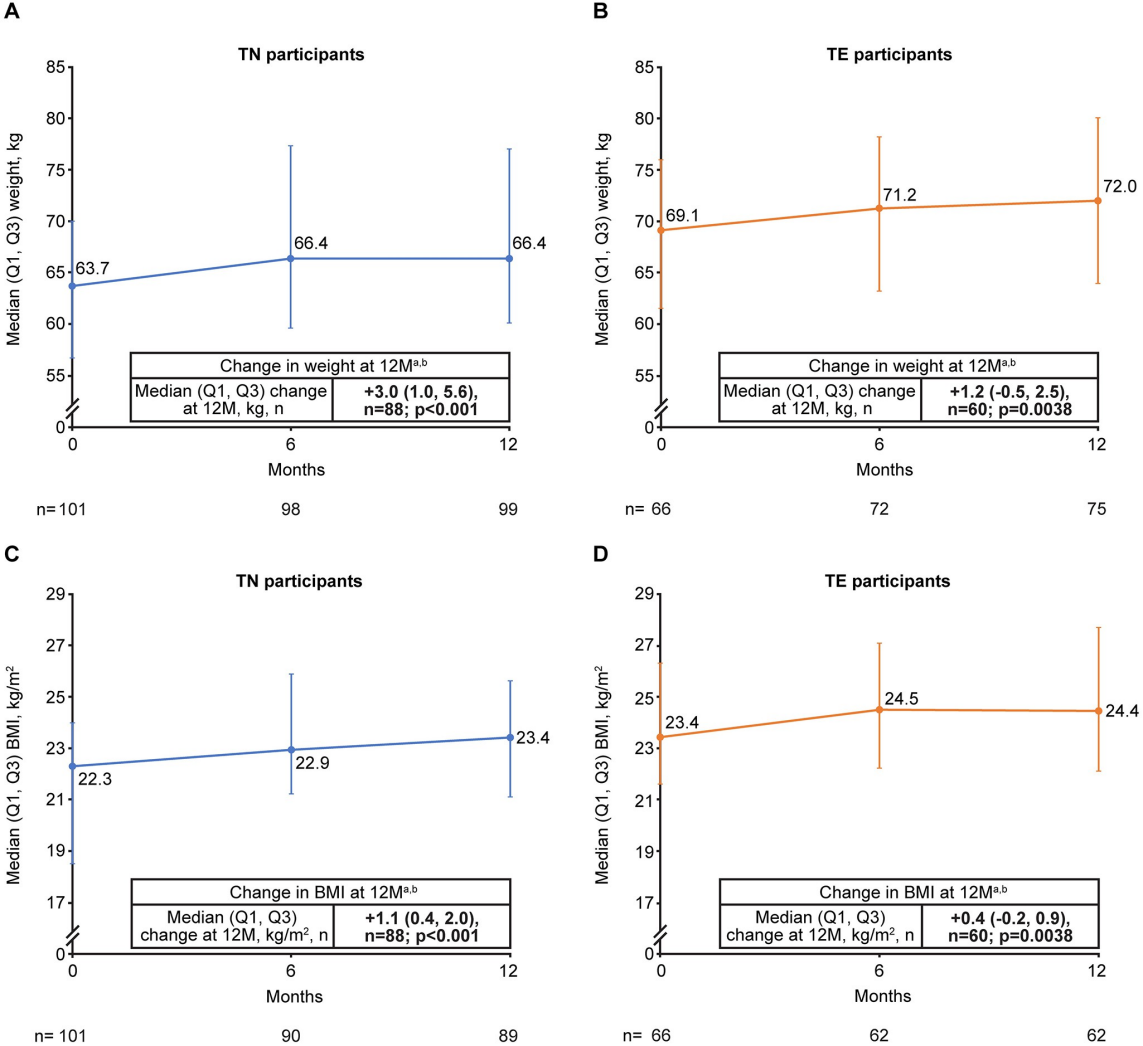
Change in weight in (A) TN and (B) TE participants and change in BMI in (C) TN and (D) TE participants from baseline to 12 months^a^. ^a^ In participants with data available at baseline and 12 months. ^b^ *p*-values calculated using the Sign test. BMI, body mass index; M, months; Q, quartile; TE, treatment-experienced; TN, treatment-naïve.

There was an increase in weight and BMI among both TN and TE participants through 12 months (Fig 4). Median (Q1, Q3) weight gain was +3.0 (1.0, 5.7) kg in TN (*p <* 0.001) and +1.2 (−0.5, 2.5) kg in TE (*p* = 0.0038) participants. Median (Q1, Q3) BMI increase was +1.1 (0.4, 2.0) in TN (*p <* 0.0001) and +0.4 (−0.2, 0.9) kg/m^2^ in TE (*p <* 0.0038) participants. Both retrospective TN and TE cohorts reported greater increases in weight and BMI compared with the corresponding prospective cohorts.

**Fig 5:**
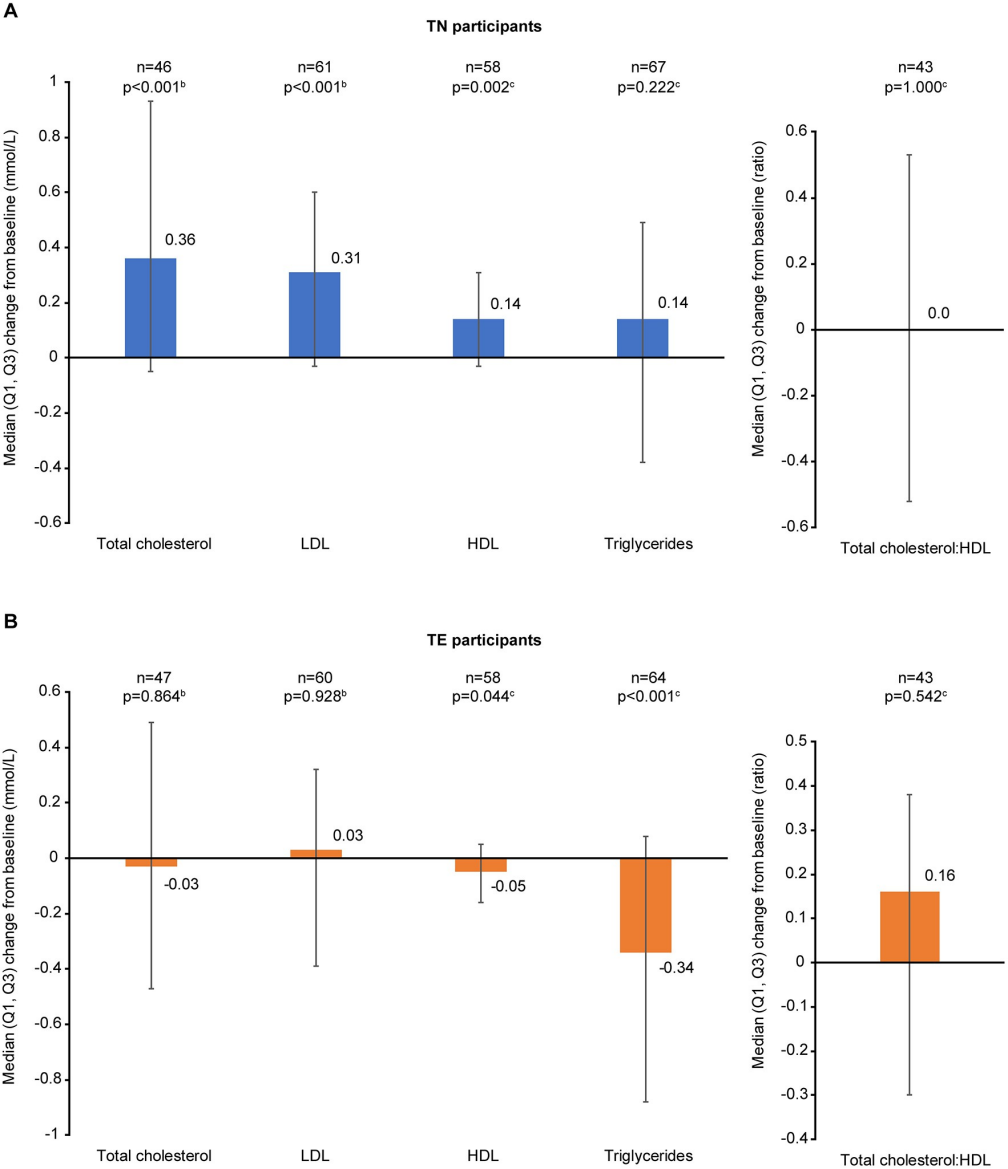
Changes in lipid levels from baseline to 12 months in (A) TN and (B) TE participants^a^. ^a^ In participants with data available at baseline and 12 months. ^b^ *p*-values calculated using the Student t-test. ^c^ *p*-values calculated using the Sign test. HDL, high-density lipoprotein; LDL, low-density lipoprotein; Q, quartile; TE, treatment-experienced; TN, treatment-naïve.

**Fig 6:**
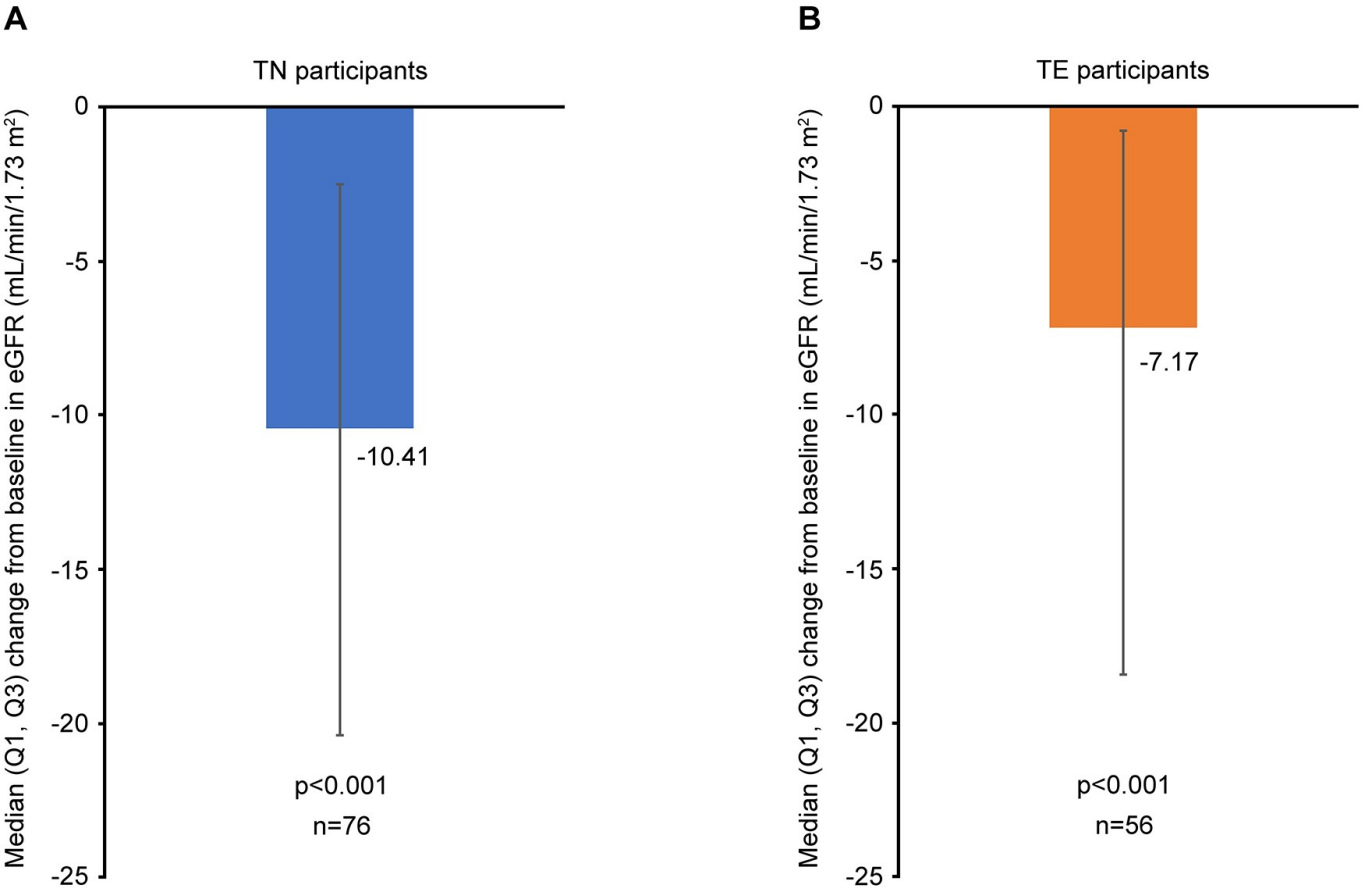
Change in eGFR levels from baseline to 12 months in (A) TN and (B) TE participants. *p*-values calculated using the Sign test. eGFR, estimated glomerular filtration rate; Q, quartile; TE, treatment-experienced; TN, treatment-naïve.

At 12 months, small but statistically significant changes from baseline in some lipid parameters were observed in TN and TE participants (Fig 5). There was also a statistically significant decrease from baseline in eGFR among both TN and TE participants (*p* < 0.001; Fig 6). At 12 months, median (Q1, Q3) glucose levels remained stable in both TN and TE participants (+0.11 [−0.22, 0.67] and 0.00 [−0.50, 0.55], respectively).

### Persistence and study-drug discontinuations

Overall, persistence on B/F/TAF was high over 12 months. At 12 months, persistence was 98% (108/110) in TN and 100% (83/83) in TE participants. Discontinuation of B/F/TAF occurred in 4% (7/200) of all participants; reasons reported for discontinuation were investigator’s decision (TN, n = 2), participant decision (TN, n = 1) and AEs (TN, n = 1; TE, n = 3) [S5 Table]. The proportion of participants discontinuing at 12 months in the retrospective and prospective cohorts was similar: 3% (5/150) and 4% (2/50), respectively.

### Patient-reported outcomes (prospective TN cohort only)

At 3 months, B/F/TAF treatment satisfaction was high in TN participants (median [Q1, Q3] HIVTSQs score: 53.0 [46.0, 57.0]). Treatment satisfaction remained high in these participants at 12 months, with a median (Q1, Q3) HIVTSQs score of 52.0 (47.5, 58.0) (S6 Table).

Median (Q1, Q3) bothersome symptom count at baseline was 3.5 (2.0, 9.0) in TN participants with data available at baseline and 12 months. A statistically significant reduction in median bothersome count was observed at 12 months in these participants (median [Q1, Q3] change: −1.5 [−4.0, 1.0]; *p* = 0.034) (S6 Table).

Median (Q1, Q3) SF-36 MCS and PCS scores at baseline were 46.7 (41.2, 52.7) and 54.7 (49.3, 56.8), respectively, in TN participants with data available at baseline and 12 months (where scores >50 indicate better than average function). At 12 months, MCS scores showed a statistically significant improvement (median [Q1, Q3] change: +2.4 [−1.7, 7.6]; *p* = 0.0386). Median PCS scores showed a trend for improvement at 12 months, although the change was not statistically significant (median [Q1, Q3] change: +2.1 [−1.0, 3.9]; *p* = 0.1442) (S6 Table).

## Discussion

These 12-month findings from the BICSTaR Japan cohort support the use of B/F/TAF in the treatment of people with HIV in Japan.

Following 1 year of treatment, B/F/TAF demonstrated high levels of virologic effectiveness and persistence, with virologic suppression observed in 93% of all participants with available data. Further, increases in both CD4 cell count and CD4/CD8 ratio indicated immunological improvements in TN participants, while immunological function was maintained in TE participants. These findings build upon results from previous clinical trials that support the efficacy of B/F/TAF in different populations of people with HIV, including women and people aged ≥65 years [15–22], and supplement existing real-world evidence for the effectiveness of B/F/TAF in routine clinical care [24–32]. The observed safety profile of B/F/TAF was consistent with that previously reported in the pooled BICSTaR cohort [33] and BICSTaR Asia cohort [34]. High levels of tolerability were reflected in minimal study discontinuations and high levels of persistence (99%) through 12 months. In prospective TN participants, PROs also indicated high treatment satisfaction and improvements in quality of life following 12 months of treatment, with fewer bothersome symptoms and improvements in mental functioning.

Increases in weight and BMI were observed in TN participants and, to a lesser extent, in TE participants following 12 months of treatment with B/F/TAF, with weight gain reported as a DRAE in five TN participants. Overall, median weight gain in the Japan cohort (TN: +3.0 kg; TE: +1.2 kg) was comparable to that reported in the pooled BICSTaR cohort (TN: +3.0 kg; TE: +1.0 kg) [33]. Weight gain has previously been described in studies of TN people with HIV taking ART [40, 41] and may, in part, represent a return to health (and a healthy bodyweight) in this population, as the catabolic effects of HIV are counteracted by treatment [42]. Similarly, switching ART regimens in virologically suppressed people with HIV has also been associated with weight gain—particularly in individuals switching away from regimens that include efavirenz or tenofovir disoproxil fumarate, which may have weight-suppressing effects [43]. Observed weight gain in the TE cohort were consistent with a pooled analysis of three randomized clinical trials in virologically suppressed Asian people with HIV, which reported a median weight gain of 1.5 kg through 48 weeks of treatment with B/F/TAF [22]. It is possible that the impact of reduced exercise and dietary changes due to the recent COVID-19 pandemic may have been a contributing factor to weight gain across TN and TE participants [44]; however, the impact of COVID-19 was not assessed during this study. The BMI of the overall population of men in Japan has displayed a slow increase over recent years, with the average BMI increasing from 23 to 24 kg/m^2^ over the past two decades [45]. The weights and BMIs reported in our study were in line with those reported in the general Japanese male population in a 2019 Ministry of Health, Labour and Welfare report (weight, 70.0 and 72.8 kg, and BMI, 23.7 and 24.7 kg/m^2^, in people aged 30–39 and 40–49 years, respectively) [46]. Additionally, although small changes in some lipid parameters were observed in our study, none were clinically significant.

A decrease in eGFR at 12 months was observed in both TN and TE participants, which was slightly more pronounced in the TN cohort. Previous studies have reported eGFR reductions in people with HIV [47, 48], and while the contributing mechanism(s) are not fully understood, inhibition of renal tubular creatinine secretion via organic cation transporter-2 has been reported with the use of certain ARTs [49]. No renal DRAEs were reported in our study, and additional analyses will be required to fully assess implications of eGFR changes.

Real-world evidence for the use of B/F/TAF specific to Japan has, until now, been very limited. Therefore, to our knowledge, our study provides the first real-world evidence of the safety and virologic effectiveness of B/F/TAF in a cohort representative of the current population with HIV in routine clinical care in Japan. Our findings are consistent with previously published data from a pooled analysis of people with HIV enrolled in BICSTaR across 12 countries [33], which reported high levels of virologic effectiveness, with 96% of all participants experiencing virologic suppression at 12 months, and improvements in immunological function in TN participants.

Our analyses are subject to the inherent limitations associated with all observational studies, including lack of randomization, selection and information bias and missing data. To reduce selection bias, eligibility criteria were well defined and retrospective participants were recruited in a consecutive manner. We numerically compared virologic suppression rates in retrospective and prospective cohorts to assess any potential for immortal time bias in the retrospective cohort that might arise from exclusion of treatment failures before study entry. We found no indication of immortal time bias with regard to either overestimation of virologic effectiveness or underestimation of AEs.

The overall study cohort was comprised of predominantly men (99%), which is largely consistent with HIV-1 incidence/prevalence estimates in Japan that have reflected a higher HIV burden in men than women [50, 51]. Consequently, the data from our analyses, including DRAEs, may not be generalizable to women with HIV. The sample size of TE participants was relatively small (n = 84), potentially negatively impacting the generalizability of results for this cohort. Additionally, the prospective TE cohort was too small (n = 8) to draw meaningful conclusions about PROs in this population. However, one strength of the study was the use of a common protocol across all BICSTaR cohorts, which enabled comparison of data from the Japan cohort with a larger and more diverse global study population. Generally, observations were similar to those of the larger multinational analyses [33]. As prospective and retrospective data were generally similar, these analyses were combined for most outcomes.

The 12-month observation period of this interim analysis is a relatively short time for evaluation of persistence, safety, and tolerability. However, our results are consistent with safety observations from previous studies of virologically suppressed people with HIV switching to B/F/TAF. One 48-week study in Asia reported minimal DRAEs and no treatment discontinuations due to DRAEs after switching to B/F/TAF [22]. In another study in Taiwan, participants reported improvements in the incidence of symptoms and their impact 48 weeks after switching to B/F/TAF [52]. The final results of the study will provide longer-term (24-month) data.

## Conclusions

B/F/TAF demonstrated high levels of virologic effectiveness and persistence, and was generally well tolerated through 12 months, with no new or unexpected safety findings. These real-world data support the use of B/F/TAF in Japanese people with HIV receiving treatment in routine practice.

## Supporting information

S1 Table Baseline demographics and disease characteristics for retrospective and prospective cohorts. (PDF)

S2 Table Reasons for initiating/switching to B/F/TAF. (PDF)

S3 Table Prevalence of key primary HIV drug-resistance mutations at baseline. (PDF)

S4 Table Virologic effectiveness at 3, 6, and 12 months (D = F analysis). (PDF)

S5 Table B/F/TAF discontinuations within the 12 months following treatment initiation. (PDF)

S6 Table Patient-reported outcomes at baseline and change from baseline at 12 months (prospective cohort only). (PDF)

S1 Fig Virologic outcomes at 12 months (M = E analysis) for the retrospective and prospective populations (A) TN and (B) TE participants. (PDF)
