# Supplementary material for: Bictegravir/emtricitabine/tenofovir alafenamide (B/F/TAF) in treatment-naïve and treatment-experienced people with HIV: 12-month virologic effectiveness and safety outcomes in the BICSTaR Japan cohort
Source: PLoS One. 2025 Jan 8;20(1):e0313338. doi: 10.1371/journal.pone.0313338 (PMC11709318; doi:10.1371/journal.pone.0313338)
Supplement: S1 Table — (PDF) [file pone.0313338.s001.pdf]

S1 Table. Baseline demographics and disease characteristics for retrospective and prospective cohorts.

	TN (n=116)		TE (n=84)	
	Retrospective (n=74)	Prospective (n=42)	Retrospective (n=76)	Prospective (n=8)
Sex,[a] n (%)				
Male	74 (100)	42 (100)	74 (97.4)	8 (100)
Female	0	0	2 (2.6)	0
Age				
Median (Q1, Q3), years	34.0 (28.0, 44.0)	34.5 (30.0, 45.0)	44.0 (38.5, 50.0)	46.5 (42.0, 58.0)
<50 years, n (%)	64 (86.5)	40 (95.2)	55 (72.4)	5 (62.5)
≥50 years, n (%)	10 (13.5)	2 (4.8)	21 (27.6)	3 (37.5)
Median (Q1, Q3) weight, kg	n=63<br>64.2 (57.3, 72.0)	n=38<br>61.5 (55.7, 68.0)	n=59<br>70.6 (62.5, 76.0)	n=7<br>64.3 (57.6, 78.0)
Median (Q1, Q3) BMI, kg/m^2	n=63<br>22.6 (19.9, 24.9)	n=38<br>21.7 (19.0, 23.2)	n=59<br>23.6 (21.6, 26.5)	n=7<br>23.1 (20.1, 24.3)
Race/ethnicity, n (%)				
Asian	73 (98.6)	41 (97.6)	76 (100)	8 (100)
White	1 (1.4)	0	0	0
Asian and White	0	1 (2.4)	0	0
Comorbidities/co-infections, n (%)				
None	21 (28.4)	11 (26.2)	18 (23.7)	0
1	23 (31.1)	10 (23.8)	13 (17.1)	2 (25.0)
2	11 (14.9)	11 (26.2)	13 (17.1)	0
≥3	19 (25.7)	10 (23.8)	32 (42.1)	6 (75.0)
Most common				
Hyperlipidemia	3 (4.1)	1 (2.4)	25 (32.9)	2 (25.0)
Neuropsychiatric	10 (13.5)	5 (11.9)	12 (15.8)	3 (37.5)
Hypertension	6 (8.1)	1 (2.4)	12 (15.8)	1 (12.5)
Diabetes mellitus	1 (1.4)	2 (4.8)	7 (9.2)	1 (12.5)
Chronic hepatitis B/C	1 (1.4) / 1 (1.4)	0 / 0	5 (6.6) / 2 (2.6)	1 (12.5) / 0
HIV-1 RNA viral load	n=71	n=41	n=67	n=8
Median (Q1, Q3), log_{10} copies/mL	4.86 (4.51, 5.27)	4.81 (4.38, 5.33)	1.28 (1.28, 1.28)	1.28 (1.28, 1.28)
<50 copies/mL, n (%)	0	0	58 (86.6)	8 (100)
>100,000 copies/mL, n (%)	28 (39.4)	15 (36.6)	3 (4.5)	0

	TN (n=116)		TE (n=84)	
	**Retrospective (n=74)**	**Prospective (n=42)**	**Retrospective (n=76)**	**Prospective (n=8)**
Median (Q1, Q3) CD4 count, cells/µL	n=70<br>290.0 (162.0, 453.0)	n=40<br>323.5 (212.0, 503.5)	n=67<br>560.0 (427.0, 713.0)	n=8<br>664.5 (453.5, 917.0)
Median (Q1, Q3) CD4/CD8 ratio	n=68<br>0.29 (0.18, 0.48)	n=40<br>0.34 (0.20, 0.48)	n=66<br>0.90 (0.60, 1.09)	n=8<br>0.82 (0.63, 1.05)
Late HIV diagnosis, n (%)	n=73	n=41		
CD4 <350 cells/µL[b]	50 (68.5)	24 (58.5)	-	-
CD4 <200 cells/µL[b]	22 (30.1)	10 (24.4)	-	-
Concomitant non-ART medications at baseline, n (%)	n=56	n=32	n=71	n=7
None	35 (62.5)	19 (59.4)	33 (46.5)	2 (28.6)
1	8 (14.3)	5 (15.6)	12 (16.9)	0
2	6 (10.7)	3 (9.4)	11 (15.5)	1 (14.3)
≥3	7 (12.5)	5 (15.6)	15 (21.1)	4 (57.1)
Median (Q1, Q3) number of previous ART regimen	-	-	2.0 (1.0, 3.0)	2.0 (1.5, 3.5)
Prior ART regimen (taken just prior to B/F/TAF), n (%)				
INSTI	-	-	45 (59.2)	4 (50.0)
NNRTI	-	-	15 (19.7)	2 (25.0)
PI	-	-	12 (15.8)	3 (37.5)
TDF	-	-	6 (7.9)	0
TAF	-	-	53 (69.7)	6 (75.0)
History of prior virologic failure, n (%)	-	-	n=76<br>2 (2.6)	n=8<br>0
Time from HIV diagnosis to B/F/TAF initiation, median (Q1, Q3) days	n=74<br>62.5 (36.0, 105.0)	n=42<br>53.5 (40.0, 100.0)	-	-

[a] Sex was defined by the individual.

[b] And/or ≥1 AIDS-defining event at baseline.

ART, antiretroviral therapy; B/F/TAF, bictegravir/emtricitabine/tenofovir alafenamide; BMI, body mass index; CD, cluster of differentiation; INSTI, integrase strand transfer inhibitor; NNRTI, non-nucleoside reverse transcriptase inhibitor; PI, protease inhibitor; Q, quartile; TAF, tenofovir alafenamide; TDF, tenofovir disoproxil fumarate; TE, treatment-experienced; TN, treatment-naïve.
