# Supplementary material for: Bictegravir/emtricitabine/tenofovir alafenamide (B/F/TAF) in treatment-naïve and treatment-experienced people with HIV: 12-month virologic effectiveness and safety outcomes in the BICSTaR Japan cohort
Source: PLoS One. 2025 Jan 8;20(1):e0313338. doi: 10.1371/journal.pone.0313338 (PMC11709318; doi:10.1371/journal.pone.0313338)
Supplement: S2 Table — (PDF) [file pone.0313338.s002.pdf]

S2 Table. Reasons for initiating/switching to B/F/TAF.

**Reason for initiating B/F/TAF**	**TN (n=116)**
Early treatment according to guidelines	114 (98.3)
Treatment as prevention	2 (1.7)
Participant's wish	1 (0.9)
**Reason for switching to B/F/TAF**	**TE (n=84)**
Simplification of ART	49 (58.3)
Participant preference	20 (23.8)
Side effect of current ART	15 (17.9)
Other	6 (7.1)

All values are shown as n (%). Reasons were predefined in the case report form. A participant may have had more than one reason for switching to/initiating B/F/TAF.

ART, antiretroviral treatment; B/F/TAF, bictegravir/emtricitabine/tenofovir alafenamide; TE, treatment-experienced; TN, treatment-naïve.
