# Supplementary material for: Bictegravir/emtricitabine/tenofovir alafenamide (B/F/TAF) in treatment-naïve and treatment-experienced people with HIV: 12-month virologic effectiveness and safety outcomes in the BICSTaR Japan cohort
Source: PLoS One. 2025 Jan 8;20(1):e0313338. doi: 10.1371/journal.pone.0313338 (PMC11709318; doi:10.1371/journal.pone.0313338)
Supplement: S3 Table — (PDF) [file pone.0313338.s003.pdf]

S3 Table. Prevalence of key primary HIV drug-resistance mutations at baseline.

	All (n=200)	TN (n=116)	TE (n=84)
≥1 primary resistance mutation, n (%)[a]			
Yes	5 (2.5)	4 (3.5)	1 (1.2)
No	64 (32.2)	58 (50.4)	6 (7.1)
Primary resistance mutations of interest, n (%)			
NNRTI	1 (0.5)	1 (0.9)	0
Y181C/I/V	1 (0.5)	1 (0.9)	0
PI	1 (0.5)	1 (0.9)	0
V32I	1 (0.5)	1 (0.9)	0
NRTI	3 (1.5)	2 (1.7)	1 (1.2)
A62V	1 (0.5)	1 (0.9)	0
T69ins	1 (0.5)	0	1 (1.2)
K70R/E	1 (0.5)	1 (0.9)	0
INSTI	0	0	0

[a] Genotype data were available for 69 participants at baseline (obtained either at the time of enrollment or from historic HIV-1 genotype tests); data were unavailable for n=131 (54 TN and 77 TE) – these participants were not considered to have primary resistance mutations.

INSTI, integrase strand transfer inhibitor; NNRTI, non-nucleoside reverse transcriptase inhibitor; NRTI, nucleoside reverse transcriptase inhibitor; PI, protease inhibitor; TE, treatment-experienced; TN, treatment-naïve.
