# Supplementary material for: Bictegravir/emtricitabine/tenofovir alafenamide (B/F/TAF) in treatment-naïve and treatment-experienced people with HIV: 12-month virologic effectiveness and safety outcomes in the BICSTaR Japan cohort
Source: PLoS One. 2025 Jan 8;20(1):e0313338. doi: 10.1371/journal.pone.0313338 (PMC11709318; doi:10.1371/journal.pone.0313338)
Supplement: S4 Table — (PDF) [file pone.0313338.s004.pdf]

S4 Table. Virologic effectiveness at 3, 6, and 12 months (D=F analysis).

	TN (n=116)	TE (n=84)
**Baseline, n**	112	75
HIV-1 RNA viral load, n (%)		
<50 copies/mL	0	66 (88.0)
95% CI	-	(78.4–94.4)
≥50 copies/mL	112 (100)	9 (12.0)
95% CI	(96.8–100)	(5.6–21.6)
**3 months, n**	104	76
HIV-1 RNA viral load, n (%)		
<50 copies/mL	85 (81.7)	72 (94.7)
95% CI	(72.9–88.6)	(87.1–98.5)
≥50 copies/mL	19 (18.3)	4 (5.3)
95% CI	(11.4–27.1)	(1.5–12.9)
**6 months, n**	103	75
HIV-1 RNA viral load, n (%)		
<50 copies/mL	97 (94.2)	72 (96.0)
95% CI	(87.8–97.8)	(88.8–99.2)
≥50 copies/mL	6 (5.8)	3 (4.0)
95% CI	(2.2–12.2)	(0.8–11.2)
**12 months, n**	100	76
HIV-1 RNA viral load, n (%)		
<50 copies/mL	90 (90.0)	72 (94.7)
95% CI	(82.4–95.1)	(87.1–98.5)
≥50 copies/mL	10 (10.0)	4 (5.3)
95% CI	(4.9–17.6)	(1.5–12.9)

At baseline, n: number of participants with a value available and analyzed within the baseline time window (no imputation). At follow-up time windows, n: number of participants with a value analyzed within the considered time window or who discontinued B/F/TAF before the considered time window and have been classified in the ≥50 copies/mL category (imputation for participants who discontinued before each time window).

B/F/TAF, bictegravir/emtricitabine/tenofovir alafenamide; CI, confidence interval; D=F, discontinuation-equals-failure; TE, treatment-experienced; TN, treatment-naïve.
