# Supplementary material for: Bictegravir/emtricitabine/tenofovir alafenamide (B/F/TAF) in treatment-naïve and treatment-experienced people with HIV: 12-month virologic effectiveness and safety outcomes in the BICSTaR Japan cohort
Source: PLoS One. 2025 Jan 8;20(1):e0313338. doi: 10.1371/journal.pone.0313338 (PMC11709318; doi:10.1371/journal.pone.0313338)
Supplement: S5 Table — (PDF) [file pone.0313338.s005.pdf]

S5 Table. B/F/TAF discontinuations within the 12 months following treatment initiation.

	TN (n=116)	TE (n=84)
B/F/TAF discontinuation within the 12 months following treatment initiation		
Yes	4 (3.4)	3 (3.6)
No	112 (96.6)	81 (96.4)
Reason for discontinuation		
Investigator's discretion	2 (1.7)	0
Participant decision	1 (0.9)	0
Adverse event	1 (0.9)	3 (3.6)

B/F/TAF, bictegravir/emtricitabine/tenofovir alafenamide; TE, treatment-experienced; TN, treatment-naïve.
