# Supplementary material for: Bictegravir/emtricitabine/tenofovir alafenamide (B/F/TAF) in treatment-naïve and treatment-experienced people with HIV: 12-month virologic effectiveness and safety outcomes in the BICSTaR Japan cohort
Source: PLoS One. 2025 Jan 8;20(1):e0313338. doi: 10.1371/journal.pone.0313338 (PMC11709318; doi:10.1371/journal.pone.0313338)
Supplement: S6 Table — (PDF) [file pone.0313338.s006.pdf]

S6 Table. Patient-reported outcomes at baseline and change from baseline at 12 months (prospective cohort only).

	TN (n=42)
**Treatment satisfaction total score (HIVTSQs)[a]**	
3 months[b]	
n	38
Median (Q1, Q3) total score	53.0 (46.0, 57.0)
12 months	
n	24
Median (Q1, Q3) total score	52.0 (47.5, 58.0)
**HIV-SI overall bothersome symptom count[c,d]**	
Baseline	
n	34
Median (Q1, Q3) overall score	3.5 (2.0, 9.0)
12 months	
n	34
Median (Q1, Q3) change from baseline	−1.5 (−4.0, 1.0)
p-value for change from baseline	0.034
**SF-36 MCS score[e,f]**	
Baseline	
n	32
Median (Q1, Q3) overall score	46.7 (41.2, 52.7)
12 months	
n	32
Median (Q1, Q3) change from baseline	+2.4 (−1.7, 7.6)
p-value for change from baseline	0.0386
**SF-36 PCS score[e,f]**	
Baseline	
n	32
Median (Q1, Q3) overall score	54.7 (49.3, 56.8)
12 months	
n	32
Median (Q1, Q3) change from baseline	+2.1 (−1.0, 3.9)
p-value for change from baseline	0.1442

[a] The HIVTSQs total score ranges from 0 to 60, with higher scores indicating higher treatment satisfaction.
[b] Treatment satisfaction data is not available for TN participants at baseline.
[c] Bothersome symptom count ranges from 0 to 20 with a higher count indicating more bothersome symptoms.
[d] Participants with HIV-SI bothersome symptom count data available at baseline and 12 months.
[e] Median scores >50 indicate better than average function.
[f] Participants with SF-36 MCS/PCS data available at baseline and 12 months.

HIV-SI, HIV-Symptom Index; HIVTSQs, HIV Treatment Satisfaction Questionnaire–Status; MCS, Mental Component Summary; PCS, Physical Component Summary; Q, quartile; SF-36, 36-item Short Form Survey; TE, treatment-experienced; TN, treatment-naïve.
