# Supplementary material for: Bictegravir/emtricitabine/tenofovir alafenamide (B/F/TAF) in treatment-naïve and treatment-experienced people with HIV: 12-month virologic effectiveness and safety outcomes in the BICSTaR Japan cohort
Source: PLoS One. 2025 Jan 8;20(1):e0313338. doi: 10.1371/journal.pone.0313338 (PMC11709318; doi:10.1371/journal.pone.0313338)
Supplement: S1 Fig — (PDF) [file pone.0313338.s007.pdf]

S1 Fig. Virologic outcomes at 12 months (M=E analysis) for the retrospective and prospective populations (A) TN and (B) TE participants.

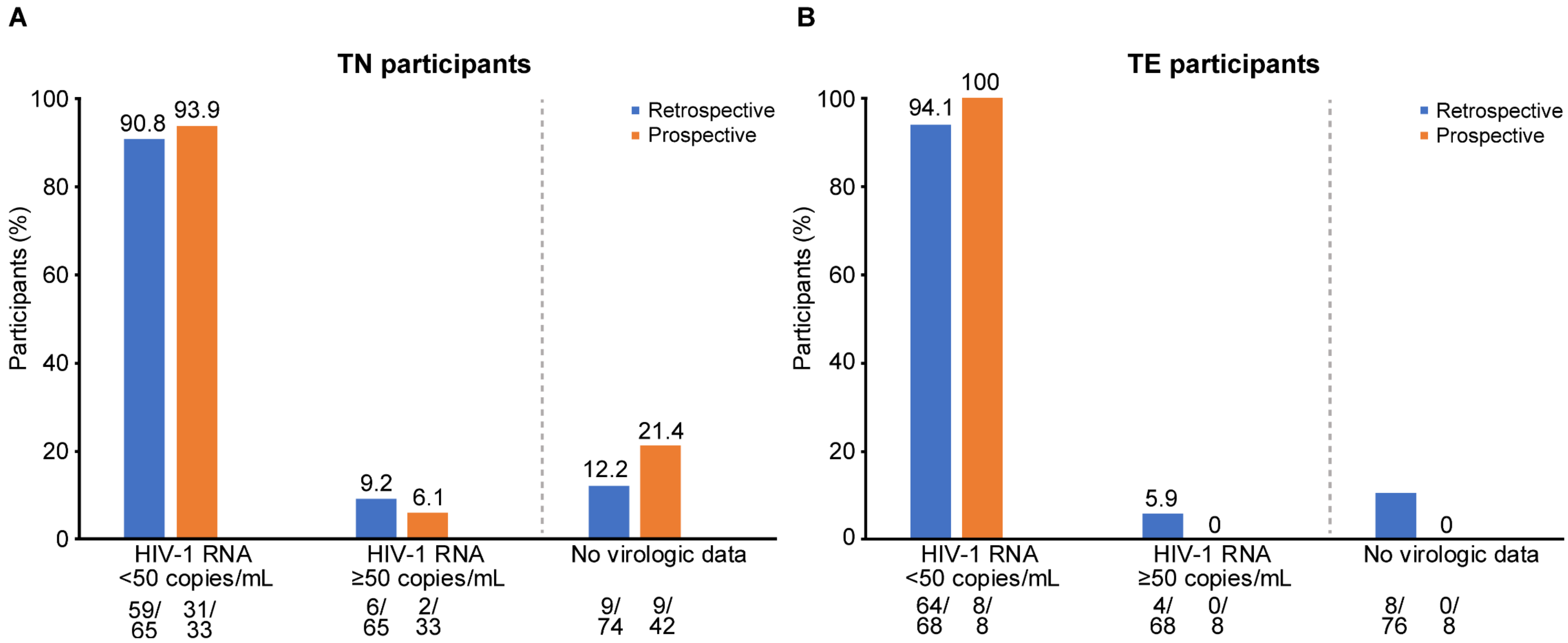

M=E, missing-as-excluded; TE, treatment-experienced; TN, treatment-naïve.
